# Photonic multiplexing techniques for neuromorphic computing

**DOI:** 10.1515/nanoph-2022-0485

**Published:** 2023-01-09

**Authors:** Yunping Bai, Xingyuan Xu, Mengxi Tan, Yang Sun, Yang Li, Jiayang Wu, Roberto Morandotti, Arnan Mitchell, Kun Xu, David J. Moss

**Affiliations:** State Key Laboratory of Information Photonics and Optical Communications, Beijing University of Posts and Telecommunications, Beijing 100876, China; Faculty of Engineering, RMIT University, Melbourne, VIC 3001, Australia; Optical Sciences Centre, Swinburne University of Technology, Hawthorn, VIC 3122, Australia; INRS-Énergie, Matériaux et Télécommunications, 1650 Boulevard Lionel-Boulet, Varennes, QC J3X 1S2, Canada

**Keywords:** integrated optics, optical computing operation, optical neural network, photonic multiplexing

## Abstract

The simultaneous advances in artificial neural networks and photonic integration technologies have spurred extensive research in optical computing and optical neural networks (ONNs). The potential to simultaneously exploit multiple physical dimensions of time, wavelength and space give ONNs the ability to achieve computing operations with high parallelism and large-data throughput. Different photonic multiplexing techniques based on these multiple degrees of freedom have enabled ONNs with large-scale interconnectivity and linear computing functions. Here, we review the recent advances of ONNs based on different approaches to photonic multiplexing, and present our outlook on key technologies needed to further advance these photonic multiplexing/hybrid-multiplexing techniques of ONNs.

## 1 Introduction

Artificial neural networks (ANNs) are mathematical models that emulate the biological brain, with their computing speed and capabilities determined by the underlying computing hardware. Mainstream electronics based on the von Neumann architecture has been widely employed, leading to significant breakthroughs in machine learning with unprecedented performance in computer vision, adaptive control, decision optimization, object identification, and more [1], [2], [3], [4], [5], [6].

However, with the ever-growing demand for processing capacity, it is clear that electronic computing alone will not be able to meet future practical requirements [7], [8], [9], [10]. Its main limitation arises from the separation of the processing unit and memory, which requires significant energy and computing power during the reading and writing of data, which leads to limited efficiency when processing ultralarge matrices. Although advanced hardware architectures, such as graphics and tensor processing units, have enabled dramatic improvements in performance, several inherent bottlenecks of electrical digital processers still exist. For example, limited by the electronic bandwidth bottleneck, the clock frequency of traditional electrical digital processors is limited to under a few GHz [11]. Further, electronic processors with higher computational power generally need a larger circuit scale and higher integration density, which will inevitably lead to high energy consumption and heat dissipation [12]. These limitations will lead to the failure of Moore’s law, thus making the realization of significantly more advanced neural networks challenging or even impossible. The development of new techniques that has the potential to overcome these limitations and achieve unprecedented computing performance is needed [13, 14].

**Figure 1:**
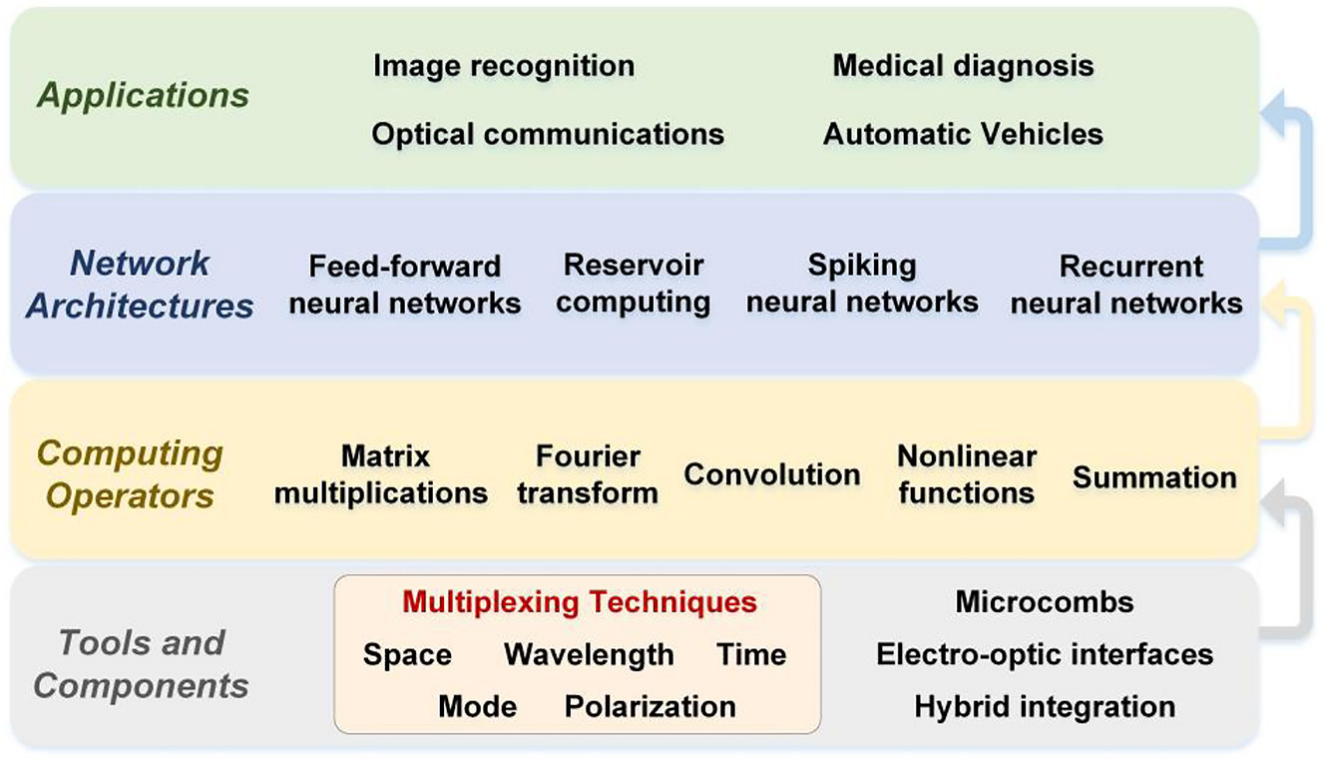
The different facets of optical neural networks.

The unique advantages of light, such as its ultrawide bandwidths of up to 10s of THz, the low propagation loss and the inherent nature of its analog architecture, make optical neuromorphic computing hardware promising to address the challenges faced by their electronic counterparts [14], [15], [16], [17], [18], [19], [20], [21], [22], [23], [24], [25]. Ultimately hybrid opto-electronic computing hardware that leverages the broad bandwidths of optics without sacrificing the flexibility of digital electronics may provide the ideal solution. Light contains multiple degrees of freedom including wavelength, amplitude, phase, mode, and polarization states, thus supporting the simultaneous processing of data in multiple dimensions via multiplexing techniques [26], [27], [28], [29], [30], [31], [32], [33], [34], [35]. Photonic techniques also have significant potential in implementing large-scale fan-in/out and weighted interconnects between neurons based on multiplexing techniques (as shown in Figure 1), which underlie the optical computing operators and further support the construction of optical neural networks (ONNs), thus simplifying the hardware architecture and addressing the demands for increased computing power in specific applications [36], [37], [38], [39], [40], [41], [42], [43].

ONNs use light as the information carrier and can simultaneously achieve the desired computing functions while propagating through specially designed dielectric structures or free space, and so the processing and storage functions are no longer separated. This passive process effectively improves the energy efficiency [44], [45], [46] and reduces the latency of ONNs – especially for approaches based on integrated platforms [47], [48], [49]. More importantly, ONNs have critical advantages for certain demanding applications such as autonomous vehicles, robotics, computer vision and other emerging fields, that require extremely rapid processing of optical and image signals. For ONNs, converting the optical and image signals into digital signals before being processed can be omitted [18], thus saving considerable time and energy.

Recently, the advances in ONNs have been reviewed from a number of different perspectives [14], [15], [16], [17], [18], [19], [20], [21], [22], [23], [24], [25], including introducing: optical field interferences for visual computing applications [18], integrated neuromorphic systems and the underlying hardware for implementing weighted interconnects and neurons [19], the training methods [21], energy consumption [22] and prospects and applications of ONNs [23]. Here, we review the most recent advances of ONNs from the perspective of the fundamental photonic multiplexing techniques that offer physical parallelism for the implementation of ONNs in contrast to previous reviews that have focused on key component and techniques [24]. These photonic multiplexing techniques include space-division multiplexing (SDM), wavelength-division multiplexing (WDM), time-division multiplexing (TDM), mode-division multiplexing (MDM), and polarization-division multiplexing (PDM). Further, we discuss the key technologies needed for the further enhancement of the computing parallelism of ONNs, which typically aim to achieve more efficient use of photonic multiplexing techniques. The paper is structured as follows. In Section 2, we survey in detail how different photonic multiplexing techniques are leveraged for the parallel signal input of vector matrix **X** and the optical weighted interconnection of the weight matrix **W** in ONNs. The typical structures of optical computing operations based on different multiplexing methods are outlined, which include matrix multiplication, Fourier transform, and convolution. In Section 3, spiking neurons and spiking neural networks based on optical multiplexing techniques are reviewed. In Section 4, we discuss how to further exploit photonic multiplexing/hybrid-multiplexing techniques in ONNs. The key technologies that further enhance the computing power through the use of photonic multiplexing/hybrid-multiplexing techniques are highlighted, including integrated optical frequency comb, integrated high-speed electronic-optical interfaces, and hybrid integration technologies.

## 2 Multiplexing techniques for optical neural networks

**Figure 2:**
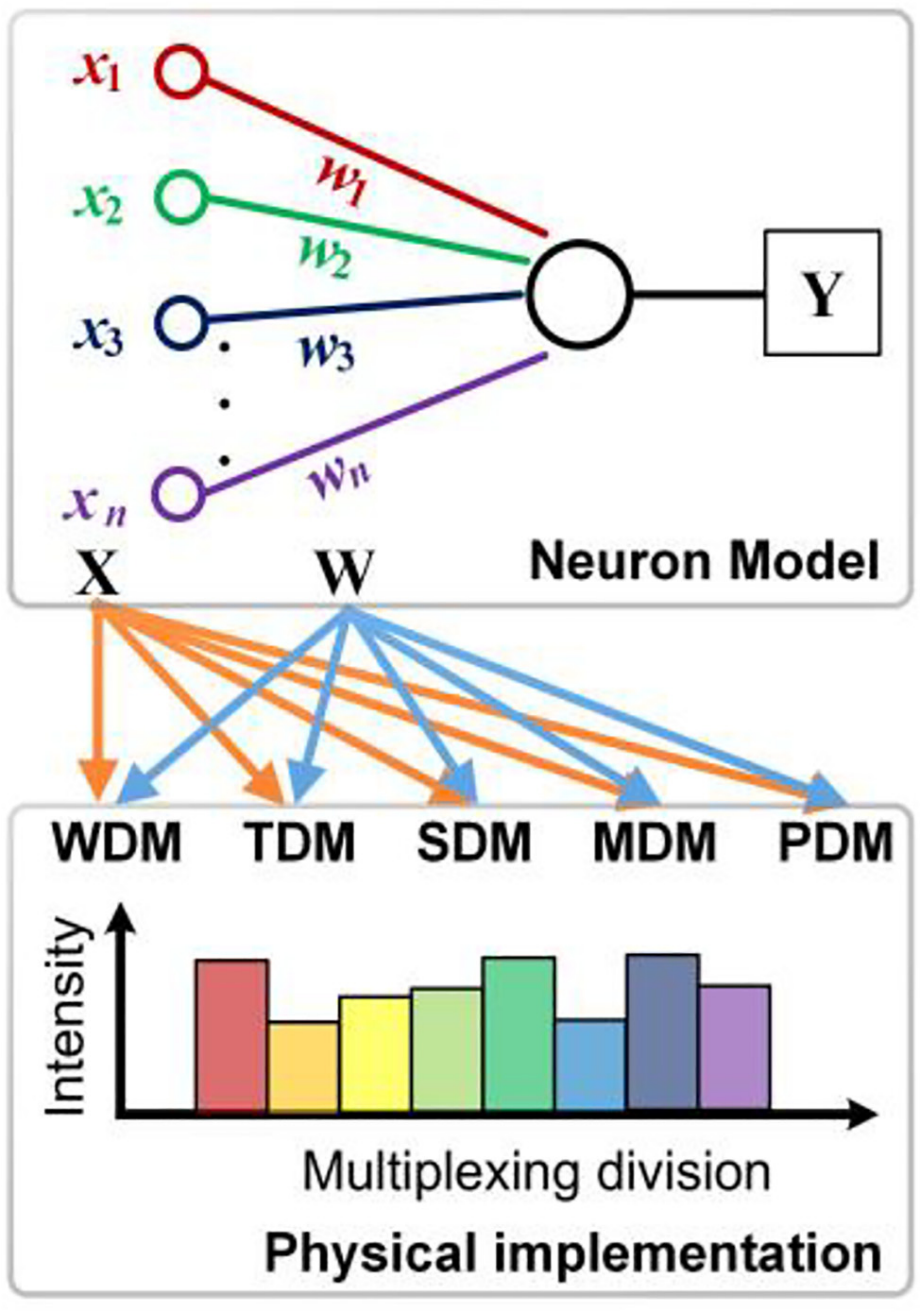
The model of a single neuron.

Neural networks typically consist of multiple layers, each of which is formed by multiple parallel neurons densely interconnected by weighted synapses. As such, the classification of ONNs can be made on the basis of a single neuron. As is shown in Figure 2, a single neuron has multiple input nodes, and the signals from different input nodes **X** are weighted via synapses **W** and summed as **Y** = **XW**. This multiply-and-accumulate operation (MAC) accounts for the majority of computations in neural networks [50, 51]. The neural network’s capacity to address complicated tasks is dictated by the scale of network (i.e., the number of neurons, synapses and layers), and thus the key to achieve maximum acceleration (using analog hardware) lies in achieving sufficiently high parallelisms and throughput to map **X** (input nodes/data) and **W** (weighted synapses) onto the practical parameters of the physical system. Analog photonics offer multiple physical degrees of freedom for multiplexing, and are thus capable of implementing large-scale fan-in/-out and synapses, with high throughput enabled by the broad optical bandwidths.

**Figure 3:**
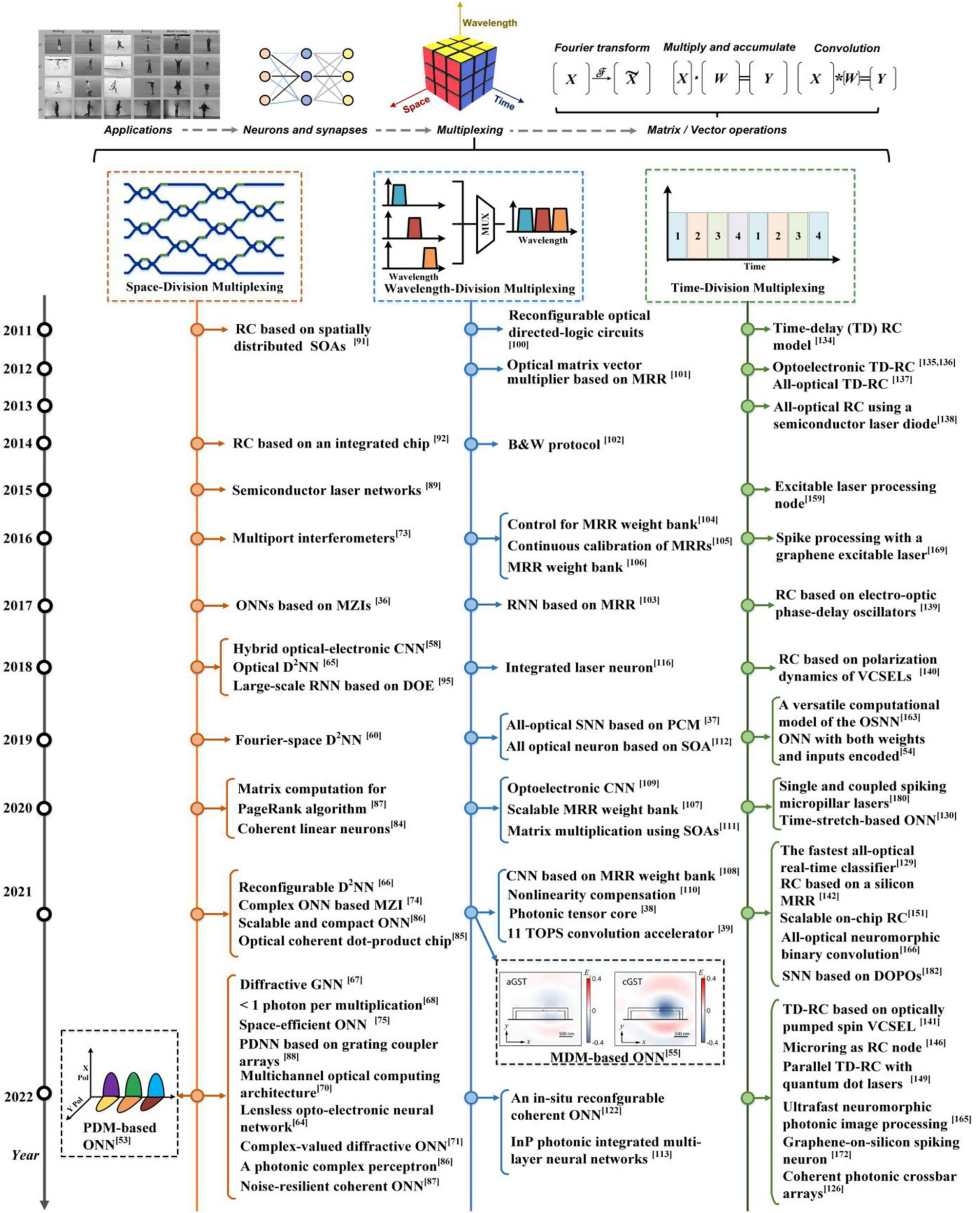
Approaches to optical neural networks using different multiplexing techniques.

Here, we classify ONNs depending on the multiplexing technique employed in terms of the input nodes **X** and the optical weighted interconnections **W** in the single neuron. We note that a single neuron can simultaneously use multiple multiplexing approaches (ideally all simultaneously to achieve the highest computing performance), as has been demonstrated for networks based on SDM [36], WDM [37], TDM [39], MDM [52], PDM [53] for **X**; and SDM [36], WDM [39], TDM [54], MDM [55], PDM [53] for **W**. Figure 3 summarizes the development history and typical structure of previously reported ONNs based on multiplexing techniques, which will be in detail introduced in this section.

### 2.1 ONNs based on space-division multiplexing

Spatial-division multiplexing is a fundamental approach to boost the computing parallelism and enhance the overall computing speed, as has been widely used in traditional digital computing systems. ONNs based on SDM feature architectures where the input nodes **X** and/or weighted synapses **W** are mapped onto the spatial division [56], [57], [58], [59], [60], [61], [62], [63], [64], [65], [66], [67], [68], [69], [70], [71], [72], [73], [74], [75], [76], [77], [78], [79], [80], [81], [82], [83], [84], [85], [86], [87], [88], [89], [90], [91], [92], [93], [94], [95], [96], [97], [98], [99]. The weighting process is achieved via manipulating the optical fields carrying data **X**, and the sum operation is achieved via constructive/destructive interference.

**Figure 4:**
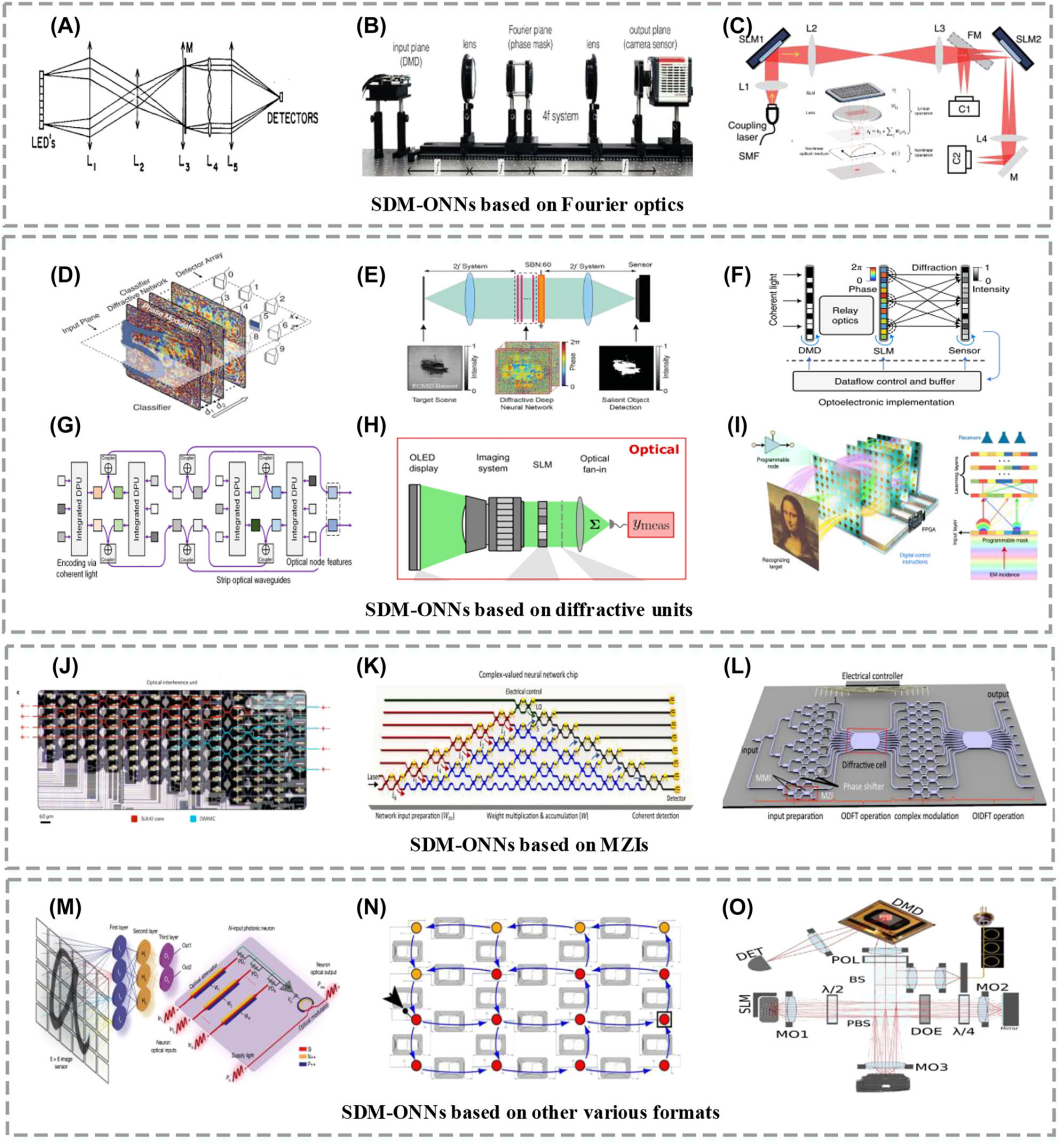
Advances in SDM-based ONNs. (A) The first optical MVM system model [57]. (B) Performing optical convolution based on the 4F system [58]. (C) A fully functioning all-optical neural network based on Fourier optics [59]. (D) An optical diffractive deep neural network [65]. (E) A Fourier-space D^2^NN [60]. (F) A reconfigurable diffractive neural network [66]. (G) A diffractive graph neural network [67]. (H) Performing optical dot products with extremely low optical energies [68]. (I) A programmable D^2^NN based on a digital-coding metasurface array [69]. (J) An all-optical neural network architecture based on MZI meshes [36]. (K) A complex neural network based on MZI meshes and the coherent detection [74]. (L) An integrated diffractive neural network [75]. (M) An integrated photonic deep neural network based on spatially distributed array of input grating couplers [88]. (N) A spatially distributed 16-node on-chip RC [92]. (O) A large scale RNN consisting of 2025 nonlinear network nodes [95].

Fourier optics [56], [57], [58], [59], [60], using a free space lens to perform Fourier transform, is a classic example of computing based on SDM and was first proposed by Bieren in 1971 [56]. Thereafter, Goodman established the model of parallel and high-speed optical discrete Fourier transforms (Figure 4A) in 1978 [57], which have been widely used to perform matrix multiplication operations [61], [62], [63]. Subsequently, the convolution operation – a more sophisticated computing operator which takes on the heaviest computational burden of convolution neural networks – was realized optically based on the 4F system (Figure 4B) by Wetzstein et al. [58]. In that system, the encoded input signals **X** go through a Fourier lens to perform a Fourier transform, and are then convolved with the convolution kernel **W** encoded onto an optimized phase mask. Later, ONNs were demonstrated with Fourier optics [59, 60], such as using Fourier lenses for the optical linear operations [59] and laser-cooled atoms with electromagnetically induced transparency for nonlinear functions (Figure 4C). Recently, Shi et al. demonstrated a novel lens-less opto-electronic neural network architecture in order to perform machine vision of the environment under natural light conditions [64]. This addresses the challenge of processing incoherent light signals by liberating lens-based optical architectures from their dependence on coherent light sources. This scheme utilized a passive optical mask designed by a task-oriented neural network to replace the lens and perform an optical convolution, thus simplifying the neural network and reducing the required complexity.

Since classical free space optics setups are relatively bulky, novel approaches such as gradient index technology, meta-surface, diffraction structures, and so on, have been exploited using SDM to achieve optical computing operators and ONNs in more compact form. In 2018, Lin et al. proposed an optical diffractive deep neural network (D^2^NN) (Figure 4D) [65], in which the information was encoded onto both the amplitude and phase of optical waves. As each pixel of the diffractive lens serves as a neuron in free space, a fully connected network involving large input nodes and neurons was realized. Following this, Dai et al. proposed a Fourier-space D^2^NN (Figure 4E) based on diffractive modulation layers [60]. The combination of diffractive optics and Fourier optics can achieve all-optical segmentation of the salient objects for the target scene after deep learning design of modulation layers, and obtained higher classification accuracy and a much more compact structure compared to real-space D^2^NN. Following this, Dai et al. [66] further optimized the diffractive neural network and proposed a reconfigurable optoelectronic neural network (Figure 4F). The key fundamental building block was the reconfigurable diffractive processing unit consisting of large-scale diffractive neurons and weighted optical interconnections. The input nodes were achieved with SDM-based spatial light modulators, with the weights tuned by changing the diffractive modulation of the wavefront. Benefiting from the high parallelism of SDM-based lenses, the proposed large-scale diffractive neural network can support millions of neurons. Soon after, Yan applied the integrated diffractive photonic computing units to the diffractive graph neural network (Figure 4G) that can perform optical message passing over graph-structured data [67], which can fulfill the recognition of skeleton-based human action. This work has inspired researchers to combine deep learning with the application-specific integrated photonic circuits design. In another example, diffractive optics were leveraged to achieve optical dot products (Figure 4H) with extremely low optical energy consumption, thus experimentally proving the advantages of photonic techniques in low-power-consumption computing [68]. Further to this, a programmable D^2^NN based on a digital-coding metasurface array [69] was proposed (Figure 4I). This D^2^NN consisted of multiple programmable physical layers, capable of dealing with image recognition, feature detection and multi-channel encoding and decoding in wireless communications by processing electromagnetic waves in free space. Recently, a novel multichannel optical computing architecture named Monet was established based on a new projection-interference-prediction framework [70]. This approach is capable of accomplishing advanced machine vision tasks such as 3D detection and moving objection detection. In this multichannel ONN, multiple inputs were projected onto a shared spatial domain and then encoded into optical fields, where optical interference and diffraction were both exploited to establish inter- and intra-channel connections, respectively. Recently, a novel diffractive all-optical neural network for physics-aware complex-valued adversarial machine learning was reported [71], based on cascaded multiple layers of (cost-effective) transmissive twisted nematic liquid crystal spatial light modulators that enabled the coupled amplitude and phase modulation of the transmitted light. This work provides the ability to manufacture and rapidly deploy large-scale photonic neuromorphic processors, with applications to adversarial and defense algorithms in the new field of complex-valued neural networks.

The interference of light represents another form of SDM based on the superposition of waves, and this can also be used to achieve optical computing [72], [73], [74], [75], [76], [77], [78], [79], [80], [81], [82], [83], [84], [85], [86], [87]. The fundamental principle is to divide coherent input light into different paths in free planar space, after which optical matrix multiplication can be achieved by appropriately designing the propagation paths of the multiply-coherent light. Typical structures to achieve interference-based computing mainly consist of Mach–Zehnder interferometers (MZIs), which are formed by beam splitters/couplers and phase shifters. In 1994, Reck et al. introduced a theoretical model for MZI-based meshes [72]. Later, Clements et al. proposed a novel universal matrix transformer in 2016 [73], with the footprint and loss further optimized. In 2017, Shen et al. proposed an all-optical neural network architecture (Figure 4J) based on a silicon photonic integrated circuit, in which 56 programmable MZIs were used for optical matrix multiplications [36]. In 2021, Zhang et al. proposed a complex neural network (Figure 4K) [74] based on coherent detection, in which information was encoded on both the magnitude and phase of light. In contrast to real-valued ONNs, this work can offer an additional degree of parallelism and achieve better performance in terms of computational speed and energy efficiency. An integrated-chip diffractive neural network (Figure 4L) was proposed in [75], where diffractive cells were introduced to implement discrete Fourier transforms. This chip is capable of performing Fourier transform and convolution operations, bringing prominent advantages in space-efficient and low-power-consuming implementations of large-scale photonics computational circuits for neural networks.

Another approach to ONNs based on optical interference is to utilize spatially distributed Mach–Zehnder modulators (MZM) to realize the physical implementation of the vector matrix **X** and the weight matrix **W** [80, 81]. Specifically, a silicon-based optical coherent dot-product chip was achieved based on a light source and spatially deployed modulator arrays in [85], where the vector matrix **X** and weight matrix **W** were mapped onto the optical fields via two push-pull configured modulators in each branch. This optically coherent dot-product chip performed computing operations in the complete real-valued domain by introducing reference light, and is capable of accomplishing sophisticated deep learning regression tasks. Very recently, a noise-resilient deep learning coherent photonic neural network was demonstrated [87], with multiple spatially deployed integrated dual-IQ electro–optic MZMs and thermo-optic MZMs that performed the vector data input and synaptic weights. Compared to coherent layouts using cascaded MZIs, the dual-IQ-modulator-based coherent photonic neural network significantly improves the noise tolerance, enabling it to realize noise-resilient deep learning at a record-high 10 GMAC/sec/axon computation rate. These dual-IQ modulator schemes can be extended to N × N neural layers via a coherent **X** bar architecture [80, 81], and in doing so support tiled matrix multiplication at high speeds [82]. Coherent photonic neurons and blocks can also be realized with single-wavelength coherent optical linear neurons [84], as well as silicon-integrated coherent neurons [83], or a complex-valued photonic perceptron [86].

The reported ONNs based SDM also include those that adopt an array of grating couplers [88], spatially distributed phase-change material (PCM) meshes [38], vertical-cavity surface-emitting lasers (VCSELs) arrays [89], and so on. In [88], an integrated photonic deep neural network (Figure 4M) with optoelectronic nonlinear activation functions was demonstrated, capable of directly processing optical waves impinging on an array of grating couplers and fulfilling image classification. A 5 × 6 array of input grating couplers distributed in free space served as input nodes to capture the image of the target object, and the weight vectors were controlled by tuning the input voltages of the PIN attenuator array. After achieving the weighted sum of the neuron inputs, the optoelectronic nonlinear response of a PN junction micro-ring modulator was used as a rectified linear unit (ReLU) which yielded the neuron’s output. This work is a significant step for the implementation of fully integrated end-to-end ONNs, and experimentally proves the advantages of ONNs for directly processing optical and image signals. In [38], the spatially distributed PCM meshes served as weighted interconnections to implement the weight addition.

Finally, SDM has also been exploited for reservoir computing (RC) [89], [90], [91], [92], [93], [94], [95], [96], [97], [98], [99] with the nodes implemented with tailored optical connection topologies [90], [91], [92], [93], [94]. In 2011, Vandoorne et al. demonstrated an integrated optical RC based on spatially distributed semiconductor optical amplifiers (SOAs) [91], whose steady state characteristics implement hyperbolic tangent nonlinear functions. Later, the authors further demonstrated that RC can be achieved on an integrated silicon photonic chip (Figure 4N) [92], which consists of passive elements such as optical waveguides, optical splitters and combiners. In 2015, Brunner and Fischer presented a spatially extended optical RC based on diffractive optical coupling. The diffractive-optical element (DOE), incorporated with an imaging lens, created coupling with the emitters of a laser array [89]. Limited only by the imaging aberration, potentially much larger network scales are possible with this diffractive coupling scheme. Later, the authors further proposed a large-scale RNN (Figure 4O) consisting of 2025 nonlinear network nodes via the DOE [95], which can individually or simultaneously realize spatial- and wavelength-division multiplexing of the output.

### 2.2 ONNs based on wavelength-division multiplexing

WDM is the prime embodiment of light’s remarkable advantages over electronics. The wide optical bands support massive wavelength channels for implementation of parallel input nodes and weighted synapses, and potentially much higher clock rates up to 10s of GHz. Specifically, the optical computing operations based on WDM can be realized by combining multi-wavelength sources with weight bands or wavelength-sensitive elements [100], [101], [102], [103], [104], [105], [106], [107], [108], [109], [110], [111], [112], [113], [114], [115], [116], [117], [118], [119], [120], [121], [122], such as micro-ring resonators (MRRs) [100], [101], [102], [103], [104], [105], [106], [107], [108], [109], [110], SOAs [111], [112], [113], and PCMs [37, 38, 114].

**Figure 5:**
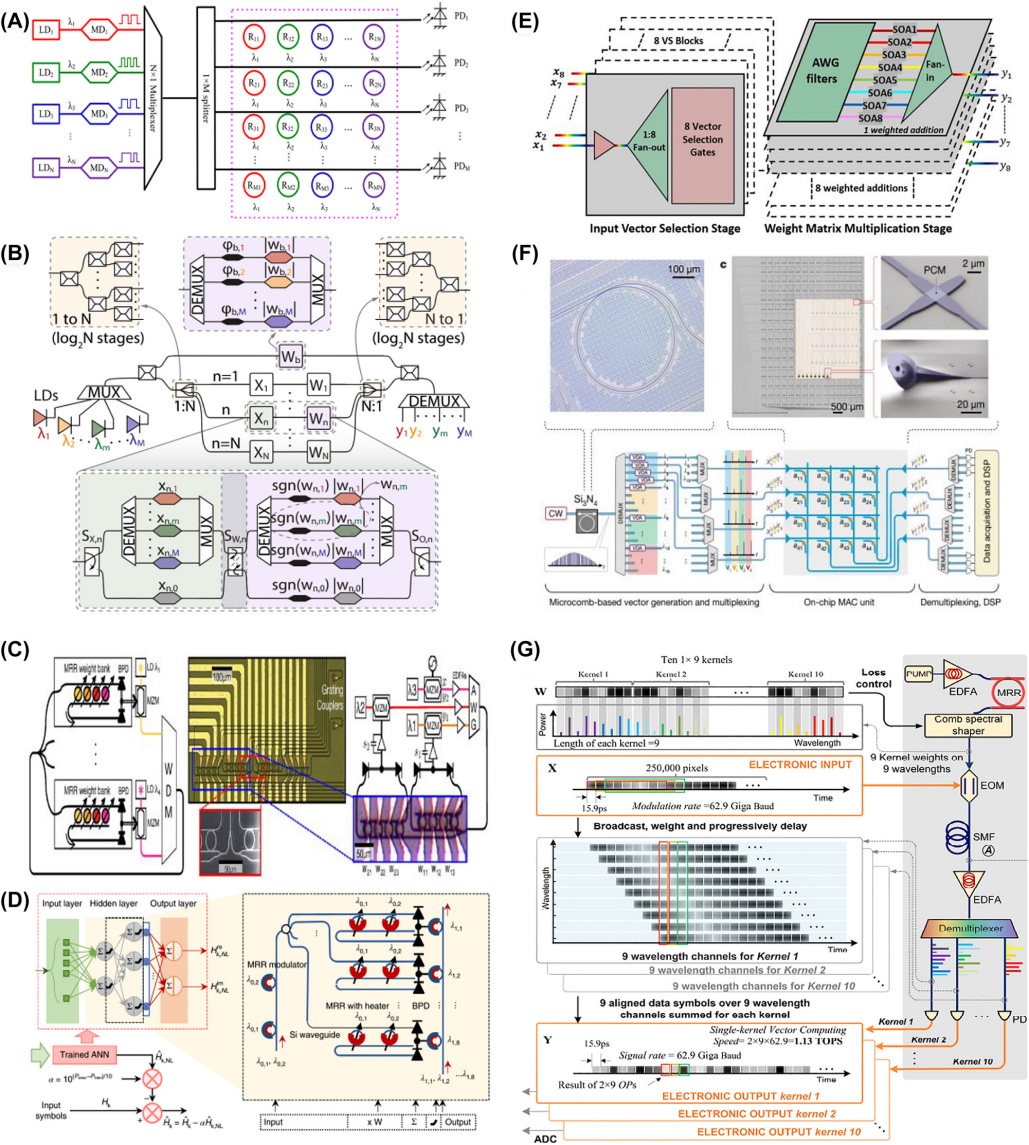
Advances in WDM-based ONNs. (A) An optical matrix vector multiplier based on WDM and MRMs [101]. (B) A programmable ONN based on WDM and coherent light [122]. (C) A continuous time RNN based on WDM and MRR weight bank [103]. (D) A WDM-based ONN for compensating the fiber nonlinearity [110]. (E) A photonic feed-forward neural network based on WDM and SOAs [111]. (F) An ONN based on WDM and PCM [38]. (G) An optical convolution accelerator based on a time-wavelength interleaving multiplexing technique [39].

In 2011, Xu et al. proposed a WDM circuit to perform incoherent summation by collecting light outputs of different wavelengths into a waveguide via a tunable MRR [100]. Soon after, Yang et al. designed an optical matrix vector multiplier (Figure 5A) that was composed of an array of cascaded lasers and modulators, wavelength multiplexers/demultiplexers, an MRR matrix and photodetectors, capable of performing 8 × 107 MACs per second [101]. The input data vector was mapped onto the optical power of different wavelengths, and the element *a*_*ij*_ of the weight vector was converted to the transmittance of the MRR at row *i*, column *j*. Such structures were further investigated in [122] (Figure 5B), in which the weight vector was added by the modulator, capable of supporting four different operations on the same photonic hardware: multi-layer, convolutional, fully-connected, and power-saving layers.

In parallel, Tait et al. proposed the broadcast-and-weight protocol in 2014 [102] and further demonstrated it with an MRR weight bank in 2017 (Figure 5C) [103]. This protocol broadcast input data onto all wavelength channels via electro–optic modulation, simultaneously weighting the replicas by controlling the power of the wavelength channels. Recently, Huang et al. implemented the WDM-based ONN with a broadcast-and-weight architecture (Figure 5D) on a silicon photonic platform [110], in which the input data of difference neurons were loaded on the multiple optical wavelengths and then multiplexed on the same optical waveguide; the interconnections between the neurons were realized by using a power splitter; the weights were applied by controlling the partial transmission of the signal via an array of MRR banks. The WDM-assisted ONN can be used to compensate for fiber’s nonlinearity, leading to an improved *Q* factor in optical communication systems.

In 2020, Indium Phosphide (InP) platforms were employed to realize a photonic feed-forward neural network (Figure 5E) [111], where SOAs were employed to simultaneously compensate for losses and achieve synaptic weights. As another alternative to achieve weighted interconnects, PCM cells can make the synaptic waveguides highly transmissive or mostly absorbing by switching phase states. In 2019, Feldmann et al. first demonstrated a spiking ONN based on PCM cells. In 2021, the authors further proposed an integrated photonic tensor core (Figure 5F) to accelerate convolution operations. The employed on-chip PCM matrix [38] can implement highly parallel matrix multiplication operations, potentially at trillions of MAC operations per second.

With the recent advances in chip-scale frequency combs, wideband and low-noise integrated optical sources are readily available, greatly expanding the potential of WDM-based ONNs. Recently, an optical convolution accelerator (Figure 5G) achieving a vector computing speed at 11.3 TOPS was demonstrated based on a time-wavelength interleaving technique [39], capable of extracting the features of large-scale data with scalable convolutional kernels. The schematic of the convolution accelerator is shown in Figure 5G. Input data was mapped onto the amplitudes of temporal waveforms via digital-to-analog converters (i.e., TDM); and the convolutional kernels’ weight matrices were mapped onto the power of microcomb lines via an optical spectral shaper (i.e., WDM). After electro–optic modulation, the input data were broadcast onto multiple wavelength channels featuring progressive time delays due to second-order dispersion of the subsequent fiber spool. By setting the progressive time delay step the same as the symbol duration of the input waveform, convolution operations between the input data and convolutional kernels can be obtained after photodetection. The convolution accelerator can be further leveraged for convolutional neural networks, which feature greatly simplified parametric complexity in contrast to fully connected ONNs.

### 2.3 ONNs based on time-division multiplexing

A key advantage of ONNs is the ultrawide bandwidths offered by optics. This yields massive numbers of wavelength channels to greatly enhance the parallelism, as introduced above for WDM-based ONNs. It also enables high data throughputs of up to 10s of Giga Baud, (corresponding to the clock rate of electronic hardware) – which requires high-speed electro–optic interfaces (i.e., modulators and photodetectors), and tailored network protocol/architecture of the input nodes employing TDM techniques [54, 123], [124], [125], [126]. Here, we review the fundamental building blocks, architectures, and recent advances of TDM-based ONNs.

**Figure 6:**
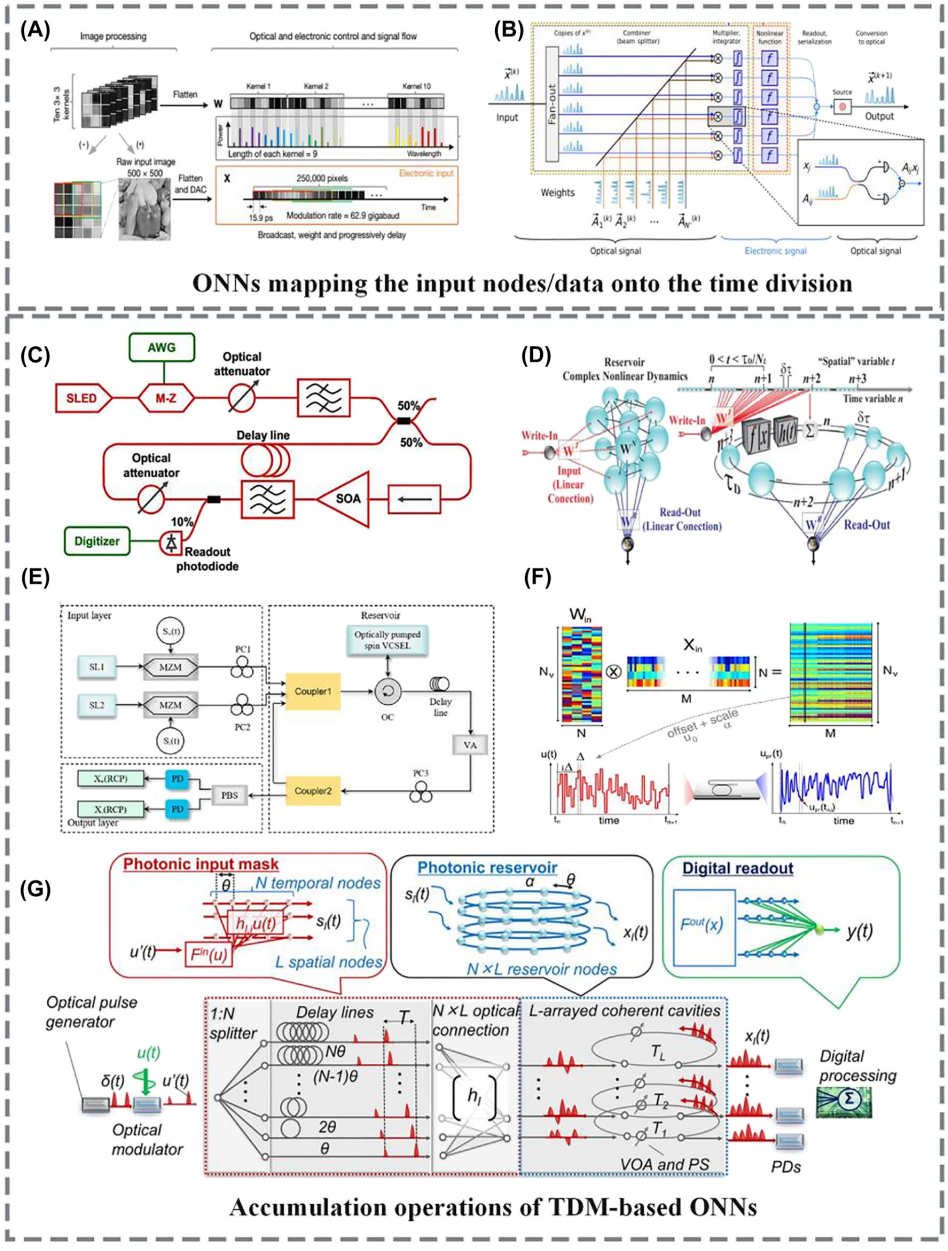
Advances in TDM-based ONNs. (A) and (B) ONNs exploiting TDM to implement large-scale fan-in/-out [39, 54]. (C) Time-delay reservoir computing (TD-RC) based on SOA [137]. (D) TD-RC based on electro–optic phase-delay oscillator [139]. (E) TD-RC based on an optically pumped spin VCSEL [141]. (F) TD-RC based on a silicon microring [142]. (G) Scalable RC on coherent linear photonic processor [151].

In [39], a high throughput exceeding 11 TOPS was demonstrated by interleaving the time- and wavelength-divisions. Here, in this work, TDM has been employed to sequentially map the input nodes/data into the time domain. Assisted by high-speed electro–optic modulators and photodetectors (>25 GHz analog bandwidth), the data rate reached ∼62.9 Giga Baud (Figure 6A). In [54], a large-scale ONN based on TDM was constructed (Figure 6B), capable of operating at high speed and very low energy per MAC. Compared to other TDM-based ONNs, this large-scale ONN encoded both the vector matrix **X** and the weight matrix **W** onto optical signals by using standard free-space optical components, allowing the weights to be reprogrammed and trained at high speed. This work shows that TDM techniques can be used to implement not only large-scale fan-in/-out architectures for input signals, but can also achieve synaptic weights, for fast, reconfigurable, and trainable ONNs. Subsequently, [126] this TDM-based optical neuron architecture was extended to an integrated photonic platform in an N × N configuration by utilizing photonic crossbar array and homodyne detection, thus achieving large-scale matrix–matrix multiplication.

The accumulation operations of TDM-based ONNs are achieved generally based on interference between signals and replicas having different delays [127], [128], [129], [130], [131], [132], [133], [134], [135], [136], [137], [138], [139]. This can be implemented using either integrated or fiber delay lines or via dispersive media. For instance, the time-stretch method based on dispersive fiber can be leveraged to implement feedforward neural networks [130], where the multiplications of input data vectors and weight matrices can be accomplished using light by stretching ultrashort pulses in the time-domain. Besides, the fiber delay lines are widely used in RC architectures.

The concept of RC, defined by Verstraeten et al. [131], was derived from the echo state network (ESN) proposed by Herbert in 2001 [132] and the liquid state machines (LSM) reported by Maass in 2002 [133]. RC is a simple and efficient machine learning algorithm suitable for processing sequential signals. It consists of an input layer, a reservoir and an output layer. The input signal is first preprocessed, and then nonlinearly mapped into a high-dimension state space by the reservoir. Afterwards, the output layer generates processed results according to the node states of the reservoir and the connection weights of the output layer. Specifically, the connection weights of the input layer and the reservoir are randomly generated and remain unchanged during the training process, while only the connection weights of the output layer are trained. In 2011, Appeltant et al. proposed an RC network that exploited a single nonlinear node with a time-delay feedback loop that yielded a large number of virtual nodes, which simplify the hardware implementation of the reservoir [134]. In 2012, Larger et al. [135] and Paquot et al. [136] first experimentally demonstrated RC using an electro–optical feedback loop with electrical gain nested, and Duport et al. implemented an all-optical RC (Figure 6C) based on a time delay feedback loop with the nonlinear function achieved with an SOA [137]. In 2013, Brunner et al. realized an all-optical RC using a semiconductor laser diode as the nonlinear node in the time delay feedback loop [138]. In 2017, Larger et al. employed electro–optic phase-delay oscillator and other traditional photonic devices to construct a photonic RC (Figure 6D) that was capable of classifying a million words per second [139]. In 2021, a novel photonic recurrent neural network with the ability to achieve time-series classification directly in the optical domain was demonstrated [129], achieving the highest computing speed for all-optical real-time classifiers at 10 Gb/s.

Recently, advances of optical RC hardware based on time delay feedback loops including those using the dual-polarization dynamics of a VCSEL [140], an optically pumped spin VCSEL (Figure 6E) [141], a silicon MRR (Figure 6F) [142], and others [143], [144], [145], [146], [147], [148], [149], [150]. In 2021, Nakajima et al. reported an on-chip RC architecture (Figure 6G) based on hybrid photonic architectures/devices [151]. The input data was spatially divided into multiple branches/temporal nodes, which were then progressively delayed via an array of delay lines and weighted by optical cross connecting units, thus achieving the input mask of RC for subsequent time-series forecasting and image classification.

TDM techniques play a significant role in a range of optical neural network structures, where they map the input signal and/or synaptic weights onto serial temporal waveforms in the optical domain, thus greatly enhancing the data input/output bandwidth. Assisted by advanced electronic digital processing techniques, TDM-based ONNs can implement a large-scale fan-in/-out of extremely high data-throughput ONNs. Furthermore, they enable the weights to be reprogrammed and trained “on the fly”, resulting in a greatly accelerated training process for the neural network. In reservoir computing, the TDM technique maps the input signals onto time-vary sequences, which simplifies the hardware implementation of the reservoir by creating a large number of virtual nodes within the time-delay loop. In spiking neural networks, the single neuron operates on discrete time spiking signals instead of continuous signals. This spiking nature enables the system to operate at ultralow powers with the ability to process temporally varying information.

### 2.4 ONNs based on mode- and polarization-division multiplexing

In addition to the approaches to ONNs introduced above, polarization- and mode-division multiplexing can also be employed to enhance the transmission capacity of optical communications [152], [153], [154] and computing parallelism of ONNs. We note that MDM and PDM are generally compatible with other multiplexing techniques, and thus can potentially lead to dramatic increases in the ONNs’ computing power. Here, we review recent advances in ONNs using those two multiplexing techniques.

**Figure 7:**
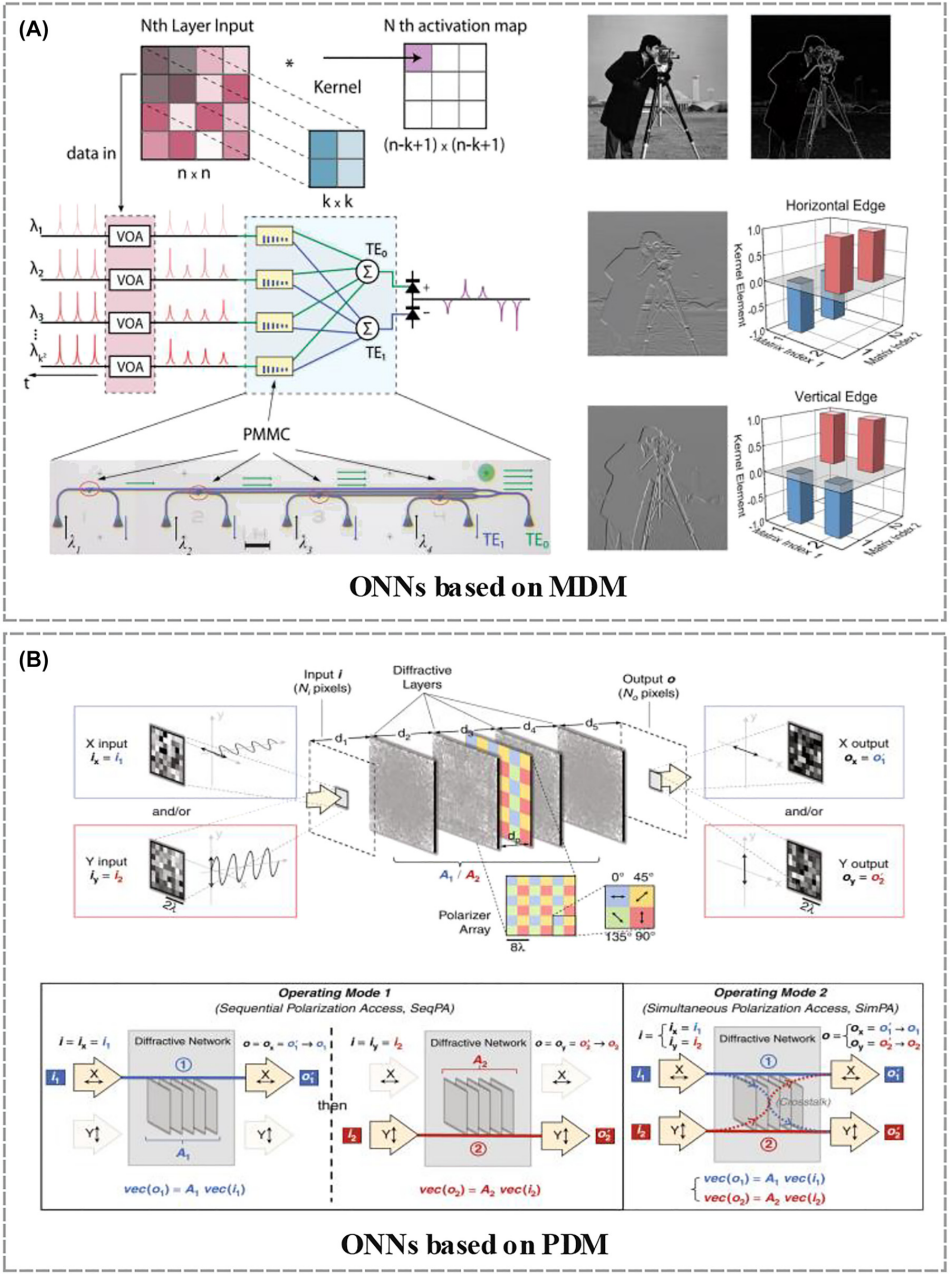
Advances in PDM- and MDM-based ONNs. (A) An ONN based on MDM and WDM [55]. (B) An ONN based on PDM and SDM [53].

In order to implement on-chip ONNs based on MDM, mode multiplexers/demultiplexers with low modal crosstalk and losses are critical. As an ideal material with optical programmability, the PCM can be employed to build programmable waveguide mode converters (other than programming the synaptic weights). Specifically, the TE_0_ and TE_1_ modes of photonic waveguides can be converted to the other via large refractive index changes of PCM Ge_2_Sb_2_Te_5_ (GST) during phase transition. Wu et al. proposed a multimode photonic convolutional neural network (Figure 7A) based on an array of programable mode converters made from PCM in 2021 [55], as shown in Figure 7A. In detail, the patches of pixels of the image were encoded onto the power of multiple wavelength channels with variable optical attenuators (i.e., WDM). The weighted data was programmed as the mode contrast coefficient of each PCM mode converter, by controlling the tunable material phases of the GST, and the transmitted results of difference modes were obtained with photodetectors.

PDM can straightforwardly double the capacity/parallelism for information transmission/processing and has been widely used in optical communications, imaging, and sensing. Recently, Li et al. proposed a diffractive ONN based on PDM (Figure 7B), which is potentially capable of fulfilling multiple, arbitrarily-selected linear transformations [53]. In this work, the computing parallelism was improved with PDM between the input and output field-of-view of the diffractive network, where the polarization states of the light will not affect the phase and amplitude transmission coefficients of each trainable diffractive feature. 2- and 4-channel optical diffractive computing based on PDM were designed for arbitrarily-selected linear transformations.

Similar to WDM techniques, both MDM and PDM result from light’s remarkable properties compared to electronics. Both can be used for to implement the input data **X** and the optical weighted interconnections **W**. Although MDM and PDM require specific components or structures to realize the required filters or mode converters and polarization devices, thus introducing additional complexity and possibly a larger footprint, they can nonetheless serve as complementary techniques to other photonic multiplexing methods, and can thus further enhance the parallelism and computing power of the system.

## 3 Multiplexing techniques for spiking neurons

Representing the third generation of ANNs [155], spiking neural networks (SNN) operate on time-discrete spiking signals instead of continuous signals. Inspired by the biological human brain neurons, artificial spiking neurons encompass binary states (active and inactive), and are only active and output spikes at firing events. The spiking characteristics of SNNs bring about enhanced noise robustness and capabilities of processing temporally varying information, enabling the great superiority of SNNs in dealing with event-based applications. Here, we survey existing structures of optical spiking neurons, one of the most fundamental components of optical SNNs, and discuss the roles of photonic multiplexing techniques in achieving them.

**Figure 8:**
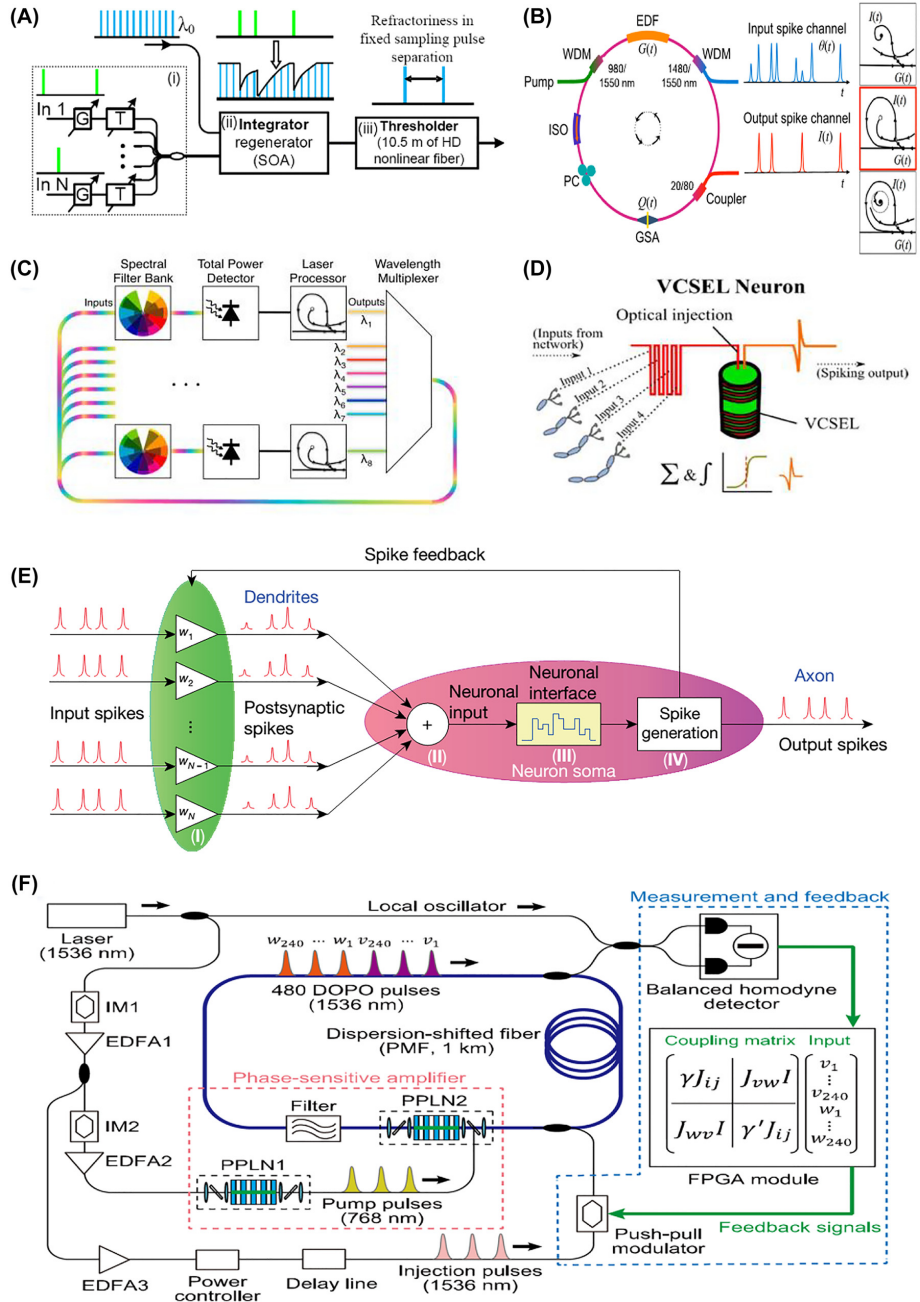
Advances in optical SNNs. (A) A spiking neuron based on SOA [156]. (B) Graphene laser-based all-optical fiber neurons [169]. (C) A spiking neuron network based on WDM [102]. (D) A spiking neuron based on VCSEL and TDM [165]. (E) A spiking neuron network based on WDM and PCM [37]. (F) Schematic diagram of a DOPO neural network based on time-domain multiplexing in a 1 km fiber-ring cavity [182].

TDM techniques were employed in most optical spiking neural networks, where the spikes/pulses are sequentially distributed in the time division. The model of spiking neurons was first proposed by Maass in [155]. Afterwards, the spiking neuron was experimentally demonstrated based on a nonlinear fiber and an SOA (Figure 8A) by Rosenbluth et al. in 2009 [156]. The SOA and the Ge-doped nonlinear fiber can achieve leaky temporal integration of a signal with thresholding functions. Later in 2011, Coomans et al. designed an optical spiking neuron based on a semiconductor ring laser and demonstrated the mechanism of utilizing a single triggering spike to excite consecutive spikes [157].

Since then, various lasers have been exploited as excitable devices for spiking neurons, such as distributed feedback lasers [158], [159], [160], [161], VCSELs [162], [163], [164], [165], [166], [167], and excitable fiber lasers [168], [169], [170], [171]. For instance, Nahmias et al. proposed a spiking neuron based on photodetectors and DFB lasers (with saturable absorbers inside), in which the input spiking signals were weighted by a tunable filter and summed by the photodetector, then drove the excitable laser to output spikes [158]. Later, they also embodied the saturable absorbers into VCSELs to form spiking neurons, utilizing the gain variation of VCSELs with input pulses at different wavelengths [118]. After that, Xiang et al. proposed a VCSEL-based SNN [163], and further presented a photonic approach for binary convolution [166]. Then they also emulated the sound azimuth detection function of the human brain based on VCSELs, in which the time interval of two spikes indicates the sound azimuth [167].

Other nonlinear optical cavities can also implement spiking dynamics [172], [173], [174], [175], [176], [177], [178], [179], [180], [181], [182], [183]. In 2016, Shastri et al. experimentally verified that graphene laser-based all-optical fiber neurons (Figure 8B) can implement spiking dynamics including consecutive spike generation, suppression of sub-threshold responses, refractory periods, and bursting behaviors with strong inputs [169]. Jha et al. proposed a spiking neuron using a graphene-on-silicon MRR, which enables spikes delivered at a high speed and improves the overall power efficiency [172]. Besides, with highly contrasting optical and electrical features between the amorphous and crystalline states, PCM can also implement spiking neurons. In 2018, Chakraborty et al. demonstrated an optical spiking neuron based on the phase change dynamics of GST embedded on the top of an MRR [114]. Soon after, Feldman et al. constructed an optical spiking neuron composed of the PCM and MRRs in 2019 [37], where the PCM unit on the ring resonator served as the excitable devices. In [165], a VCSEL-based spiking neuron with integrate-and-fire capability was demonstrated (Figure 8D), achieving power summation of multiple fast input pulses. In [182], a spiking neuron network based on a degenerate optical parametric oscillator was constructed (Figure 8F). It consists of a fiber-ring cavity and opto-electronic feedback system, which could accommodate more than 5000 time-domain multiplexed pulses in the 5-μs round-trip time.

The WDM technique has also played a significant role in achieving photonic SNNs. The optical pulses encoded on to different wavelengths are inherently transmitted without crosstalk, and the weighted optical signals from different nodes can be detected/summed via photodetectors. In 2014, Tait et al. proposed an optical SNN (Figure 8C) based on the WDM technique [102], supporting large-scale parallel interconnections among high-performance optical spiking neurons (as introduced in Section 2.2). The following year, Nahmias et al. presented a method to cascade DFB spiking neurons into a large-scale network [159], where the capacity of each waveguide was boosted by WDM. Afterwards, Feldman et al. demonstrated a WDM-based optical SNN (Figure 8E), with input spikes at different wavelengths multiplexed and integrated via post-synaptic spiking neuron [37].

Compared to continuous-valued ONNs, spiking ONNs are more similar to the intuitive model of biological brains. While current research mostly focuses on building high-performance spiking neurons, the network structure, data fan-in/out, and hardware integration remains an unsolved puzzle.

## 4 Outlook

The key to neuromorphic photonic computing hardware is to achieve a sufficiently large parallelism in order to map the input nodes and synapses onto physical parameters, as the optical systems are analog, as well as to boost the overall computing speed in cooperation with high-speed electro–optic interfaces. Although significant advances have been made in neuromorphic optics, the unique advantage of optics, such as the ultralarge bandwidths and multiple dimensions for multiplexing, have yet been fully realized. Extensive progress remains to be made in terms of collectively combining existing techniques to develop devices tailored for ONNs, especially in terms of key components and integration platforms, optical computing operators/algorithms, and electro–optic hybrid logics/architectures, in order to boost the computing performance of optics, making them comparable with, and ultimately enabling them to partially replace, their electronic counterparts.

The key components and integration platforms for ONNs are mainly centered on those that are critical to implementing multiplexing techniques. SDM is implemented with massive optical devices (both passive and active) densely integrated onto a single chip, which requires: low propagation loss waveguides and crossings, such as ∼1 dB/m achieved by SiN platforms using the Damascence reflow process [184]. Also important are heterogeneous integration techniques that enable compact footprints for computing cores involving active (i.e., gain media or light sources), photodetectors, modulators, and passive computing cores (such as MZI arrays) [185], [186], [187]. Finally, multilayer photonic circuits exploiting the vertical dimension of integration, other than planar circuits [188, 189] can also be exploited.

WDM is implemented mainly through the use of optical frequency combs that provide a large number of evenly spaced wavelength channels, and spectral shapers to manage the wavelength channels. Microcombs generated via parametric oscillation in high-*Q* micro-resonators, are promising optical frequency comb sources, as they offer a large number of wavelengths in integrated platforms. Significant advances have been made in microcombs, leading to a wideband, compact, high-energy-efficiency (even battery driven operation), turnkey, and mass-producible comb sources for WDM-based ONNs [190], [191], [192], [193], [194], [195], [196], [197], [198], [199], [200].

TDM is implemented based on high-speed electro–optic interfaces including modulators and photodetectors, which communicate with external electronics (such as analog-to-digital converters, digital-to-analog converters, and memories). A diverse range of integrated platforms, including lithium niobate (LiNbO_3_) [201], hybrid silicon and LiNbO_3_ [202], thin-film LiNbO_3_-on-insulator (LNOI) [203], InP [204], and hybrid silicon polymer [205], have readily demonstrated these high-speed interface devices.

PDM and MDM are additional dimensions of optics for multiplexing, and they can greatly scale the parallelism of ONNs as they can operate together with other multiplexing techniques. The key to construct PDMs or MDM-based ONNs lies in achieving on-chip polarization/mode sensitive devices to offer suitable fan-in/-out, such as the dual-polarization LNOI modulator that can utilize two polarization states [206], and the PCM cells capable of switching supported optical modes [55]. In addition, while multiplexing techniques address the mapping of neurons’ input nodes and synapses, the nonlinear functions, albeit demonstrated in [59], remains challenging for integrated platforms. They can be potentially achieved via either highly-nonlinear optical materials/structures, or electro–optic devices [88] employed in the interfaces of ONNs.

**Figure 9:**
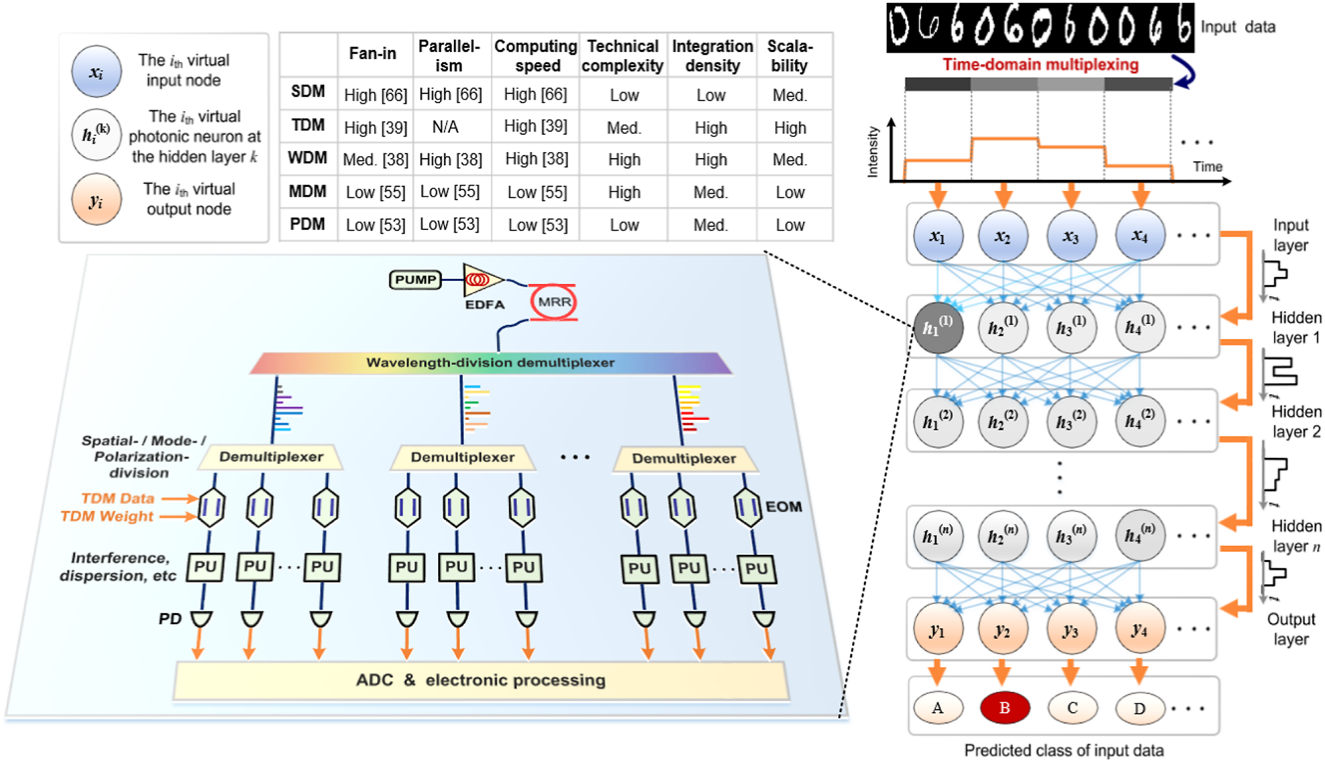
Schematic of a photonic neuron using hybrid multiplexing techniques. EOM, electro–optical modulation; PU, processing unit; PD, photodetector; ADC, analog-to-digital convertor.

In Figure 9 we compare the different multiplexing techniques, indicating their unique advantages.

(a) SDM is the most widely used technique; both in optics and electronics, where reconfigurable diffractive neural networks based on SDM [66] have achieved 2.20 million fan-in nodes of the input signal **X**, with a computing speed of 240.1 tera-operations per second, which is the highest parallelism so far in optics. SDM can also be implemented with passive optical components, resulting in low complexity, although limited by the integration density achievable with nanofabrication. SDM-based ONNs using Fourier lenses, gratings, cameras/sensors or other optical devices have critical advantages for directly processing optical data/sequences for demanding applications to autonomous vehicles, robotics, and computer vision, thus saving considerable time and energy [18]. SDM is a key technique for realizing optical neuromorphic hardware, and will likely remain the most popular approach.
(b) TDM is implemented with high-speed electro–optical interfaces and external digital-to-analog converters, and so with a sufficiently large memory, the fan-in is theoretically unlimited. TDM is a serial method, and so although it cannot boost the computing parallelism, it is attractive for handling the input and output of data and weights, since it offers a high throughput of up to tens of Giga Baud. By utilizing a 62.9-gigabaud digital-to-analogue converter to map the input vector **X** onto serial temporal waveforms in the optical domain, an optical convolutional accelerator based on TDM achieved a computing speed of 11 tera-operations per second [39]. Benefiting from mature electronic technologies, TDM can also achieve a high integration density.
  Another major advantage of TDM techniques is that they lend themselves much more readily to scaling the network in depth to multiple hidden layers. Most ONNs only achieve a single neural layer, as well as implementing their nonlinear functions in the electronic domain. Using TDM techniques, multilayer ONNs can be constructed to accomplish more challenging functions by iteratively invoking the single optical computing unit, and so TDM-based ONNs have better scalability. For reservoir computing and spiking neural networks, TDM methods help simplify the hardware and significantly reduce the power consumption.
(c) WDM, MDM and PDM based methods further exploit the advantages of optics, and are complementary to SDM approaches. WDM is one of the most direct and effective approaches to enhance the parallelism of photonic neuromorphic hardware. With the advances in hybrid integration of soliton microcombs, integrated photonic hardware accelerators that support more than 200 individual wavelengths have been reported [38], capable of operating at speeds of 1012 MAC operations per second. Hybrid integration enables WDM-based integrated photonic hardware to achieve a high technical complexity. Similar to WDM, MDM, and PDM both involve additional components to further enhance the system parallelism and thus computing power.

Finally, we note that future neuromorphic photonic processors will likely make use of all of the multiplexing techniques supported by optics, simultaneously. Figure 9 shows a schematic of a photonic neuron using hybrid multiplexing techniques. It uses TDM to achieve the data and weight input/output, and ideally uses all multiplexing methods to boost the parallelism. The processing unit (PU) can be implemented in the form of interferometers or dispersive media in order to achieve the greatest range of computing functions. The neurons can then be densely integrated into arrays to form a full neural network for specific machine learning tasks.

While analog optics features potentially much higher computing power and energy efficiency, they are inherently limited in terms of flexibility and versatility in contrast to digital electronics based on Von Neumann structures with distributed processors and memories. As such, hybrid opto–electronic neuromorphic hardware is a promising solution that leverages the advantages of both optics and electronics, where optics undertakes the majority of specific computing operations while electronics manages hardware parameters and data storage. Under such architectures, the optical computing cores serve as callable modules embedded in external electronic hardware, with the data rate and analog bandwidths matching each other. It is optimistically expected that, with more categories of optical computing operations, algorithms and architectures being demonstrated, ONNs can serve as a universal building block for diverse machine learning tasks [200, 207], [208], [209], [210], [211], [212]. With the dramatically accelerated computing speed brought about by the hybrid opto–electronic computing architecture, much more complicated neural networks can be enabled, potentially leading to revolutionary advances in applications such as automated vehicles, real-time data processing, and medical diagnosis.

## 5 Conclusions

Photonic multiplexing techniques have remarkable capacity for implementing the optoelectronic hardware that is isomorphic to neural networks, which can offer competitive performance in connectivity and linear operation of neural network. In this review, we have presented typical architectures and the recent advances of ONNs that utilize different photonic multiplexing/hybrid-multiplexing techniques involving SDM, WDM, TDM, MDM, and PDM to achieve interconnection and computing operations. The challenges and future possibilities of ONNs are also discussed.
